# Legal Pregnancy Interruption due to Sexual Violence in a Public Hospital in the South of Brazil

**DOI:** 10.1055/s-0042-1755457

**Published:** 2022-11-29

**Authors:** Vitoria Finger Trapani, Otto Henrique May Feuerschuette, Alberto Trapani Júnior

**Affiliations:** 1 Universidade do Sul de Santa Catarina, Tubarão, SC, Brazil; 2 Universidade Federal de Santa Catarina, Florianópolis, SC, Brazil

**Keywords:** abortion, sexual violence, legal abortion, women's health, health service, abortamento, violência sexual, aborto legal, saúde da mulher, serviço de saúde

## Abstract

**Objective** To analyze the cases of all women who attend to a service of legal termination of pregnancy in cases of sexual violence in a public reference hospital and to identify the factors related to its execution.

**Methods** Cross-sectional observational study with information from medical records from January 2014 to December 2020. A total of 178 cases were included, with an evaluation of the data referring to the women who attended due to sexual violence, characteristics of sexual violence, hospital care, techniques used, and complications. The analysis was presented in relative and absolute frequencies, medians, means, and standard deviation. Factors related to the completion of the procedure were assessed using binary logistic regression.

**Results** Termination of pregnancy was performed in 83.2% of the cases; in 75.7% of the cases, the technique used was the association of transvaginal misoprostol and intrauterine manual aspiration. There were no deaths, and the rate of complications was 1.4%. Gestational age at the time the patient's sought assistance was the determining factor for the protocol not being completed. Pregnancies up to 12 weeks were associated with a lower chance of the interruption not occurring (odds ratio [OR]: 0.41; 95% confidence interval [CI]: 0.12–0.88), while cases with gestational age > 20 weeks were associated with a greater chance of the interruption not happening (OR: 29.93; 95%CI: 3.91–271.50).

**Conclusion** The service studied was effective, with gestational age being the significant factor for resolution.

## Resumo

**Objetivo** Analisar os casos de todas as mulheres que buscaram o serviço de interrupção legal da gestação em casos de violência sexual em um hospital público de referência, assim como identificar os fatores relacionados à realização do procedimento.

**Métodos** Estudo observacional transversal com informações de prontuários de janeiro de 2014 a dezembro de 2020. Foram incluídos 178 casos, com avaliação dos dados referentes às mulheres atendidas, características da violência sexual, atendimento hospitalar, técnicas utilizadas e complicações. A análise foi apresentada em frequência relativa e absoluta, medianas, médias e desvio padrão. Fatores relacionados à realização do procedimento foram avaliados por meio de regressão logística binária.

**Resultados** O procedimento de interrupção da gravidez foi realizado em 83,2% dos casos atendidos. Em 75,7% dos casos, a técnica utilizada foi a associação de misoprostol por via vaginal e aspiração manual intrauterina. Não houve óbitos e a taxa de complicações foi de 1,4%. A idade gestacional no momento da busca pela interrupção legal da gravidez foi o fator significativo pelo qual o protocolo não foi concluído. Gestações de até 12 semanas foram associadas a uma menor chance de não ocorrer a interrupção (odds ratio [OR]: 0,41; intervalo de confiança [IC] 95%: 0,12–0,88), enquanto casos com idade gestacional > 20 semanas foram associados a uma maior chance de não ocorrer a interrupção (OR: 29,93; IC95%: 3,91–271,50).

**Conclusão** O serviço estudado foi efetivo, sendo a idade gestacional o fator significativo para a resolutividade.

## Introduction

Sexual violence is a humanitarian and public health issue, as the countless psychological and physical damage resulting from the sexual abuse remains with the victim after the crime. 1 The high incidence of cases of sexual violence brings to the health service patients who need assistance for possible physical and psychological damage and prophylaxis against sexually transmitted infections and pregnancy. 2 Furthermore, regarding psychological damage, it is common that the victims present anxiety and post-traumatic stress disorder, which interfere in both the individual and the collective spheres, affecting the interpersonal relationships and daily lives of the victims, also having an economic impact. 3

In 2009, a modification in the Brazilian Civil Code declared rape as a crime against sexual liberty and dignity. 4 In its article 213 (in the wording given by Law No. 12,015), rape is defined as: to constrain someone, through violence or serious threat, to have a carnal conjunction or to practice or allow another libidinous act to be performed with them. 5

Studies indicate that almost 90% of the cases of sexual violence go unreported. Deficiency, absence, or inadequate disclosure of the care network contributes to underreporting and health problems. 4 5 6 7

Among the consequences of sexual violence, pregnancy stands out due to the complexity of the psychological, social, and biological reactions it determines. Unwanted or forced pregnancy is seen as a second type of violence, intolerable for many women. 2

Women in a situation of pregnancy resulting from sexual violence, as well as adolescents and their guardians, must be informed about the legal alternatives regarding the possibilities of care in health services. It is the right of these women and adolescents to be informed of the possibility of a legal termination of pregnancy (LTP). 8

The Brazilian penal code of 1940, in its article 128, says that abortion caused by a doctor is not punishable if the pregnancy is due to rape and is preceded by the consent of the pregnant woman or of her guardian, in the case of a minor or incapable person; however, the concept of abortion was not defined in terms of gestational age. 5 In the English language and in some countries the procedure is called LTP and not abortion, and the gestational age is not limited.

Although Brazilian law has made it possible to voluntarily terminate pregnancy due to rape since the 1940s, access to health services has not been regulated for nearly 50 years. 9 In 1989, the Municipal Government of the city of São Paulo implemented the first service to assist women victims of sexual violence. 10 To have access to LTP, the woman should present a copy of the Police Report (PR) and the expert report of the Legal Medical Institute (LMI). 8 The national regulation for LTP took place in 1999, with the launching of the technical norm Prevention and Treatment of Diseases Resulting from Sexual Violence against Women and Adolescents, which stimulated and regulated the structuring of services. Updated in 2005 and in 2011, the norm exempted women from submitting a PR or LMI report; the written consent of the woman and the evaluation of an interdisciplinary team would be enough. 8

However, this access is not yet widespread and not well-known across the country. There are few services that offer the LTP resulting from sexual violence program. In the state of Santa Catarina, for example, only four public institutions are referenced by the State Health Department to have an active program. 11 12

Linked to this, the problem of abortions considered to be unsafe, when they are not performed in a medical appropriate environment or by a suitable professional, must be recognized. 13 According to the World Health Organization (WHO), ∼ 45% of abortions in the world are performed unsafely. 14 In Brazil, abortion complications are the fourth cause of maternal mortality. Between 2006 and 2019, according to the Mortality Information System (MIS), 1,059 women had abortion registered as the underlying cause of death. 15 We must also consider that many of the deaths due to abortion complications are underreported, mainly because they are performed in illegal clinics. 16 17

The care of a woman who wants to terminate a pregnancy that resulted from sexual violence, according to Brazilian law, does not depend on a judicial or police process. In the health institution, five terms are required: the free and informed consent term, the liability term signed by the victim and/or by her legal representative, the detailed report term of the woman and/or her legal representative, a technical opinion, signed by a physician, attesting the compatibility of the gestational age with the date of the sexual assault, and the term of approval of the procedure for termination of the pregnancy, signed by the multidisciplinary team. 8 18

The present study aimed to analyze the profile of the women who sought a public service for abortion in case of sexual violence, as well as their results, to help in the construction of policies and practices for health assistance to women.

## Methods

This is a cross-sectional observational study; the data has been extracted from medical records of women who sought assistance for LTP resulting from sexual violence at the Hospital Universitário Polydoro Ernani de São Thiago of the Universidade Federal de Santa Catarina (HU-UFSC, in the Portuguese acronym), Florianópolis, state of Santa Catarina, Brazil, as a source of information, during the period from January 2014 to December 2020. The study was submitted and approved by the Research Ethics Committee (REC) of the Universidade do Sul de Santa Catarina (UNISUL, in the Portuguese acronym) under the number 4.193.711.

All women who sought the LTP service due to sexual violence were included in the survey. The adopted protocol follows the definitions of the technical norms of the Brazilian Ministry of Health and defines as abortions pregnancies terminated up to 20 weeks. 18

Regarding the sexual violence suffered, the following data were collected: city and date of occurrence, use of emergency contraception, previous police report about the violence, medical care after the rape, type of aggressor, number of aggressors, type of intimidation, and place of violence.

Regarding the LTP, the following data were collected: date of the visit to the hospital, previous attempt at unsafe abortion, search for a previous hospital service, complaint to the police authority at the time of the search for the LTP, time between the visit to the hospital and hospitalization, days of hospitalization, performance or not of the procedure, method used, and observed complications.

Regarding information about the right to LTP in case of rape, it was asked how the patient knew about the right to interrupt the pregnancy and the source regarding the referral service.

Data were entered into Microsoft Excel (Microsoft Corporation, Redmond, WA, USA) and exported to SPSS PASW Statistics for Windows 18.0 (SPSS Inc., Chicago, IL, USA). They were analyzed and described as relative and absolute frequencies or by measures of central tendency and data dispersion (medians, means, and standard deviations [SDs]).

In a second step, the difference between the dependent variable of performing the interruption procedure or not and the independent variables was tested through the association measure of the odds ratio (OR) with the respective 95% confidence intervals (95%CIs), through binary logistic regression. The level of significance established was *p* < 0.05.

## Results

**Fig. 1:**
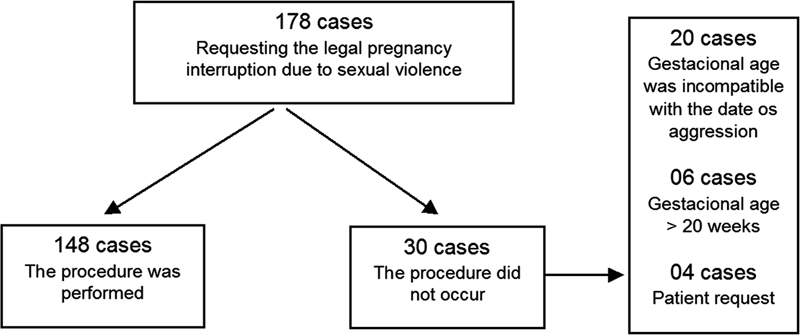
Study flowchart.

From January 2014 to December 2020, 178 women who sought the HU-UFSC requesting the procedure for LTP due to sexual violence were found. After stability in the initial 3 years of the study, between 2016 and 2019 there was an average increase of 62.3% of cases per year. In 2020, there was a 26.5% reduction in visits by the LTP group ( Fig. 1 ).

**Fig. 2:**
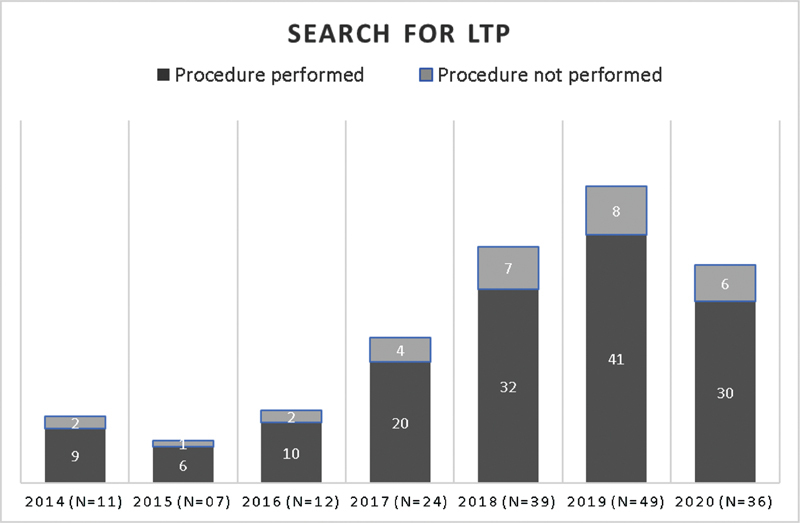
Distribution of patients who sought the service for legal termination of pregnancy due to sexual violence, according to the year and the procedure being performed or not.

Among the 178 cases in which the LTP protocol for sexual violence was initiated, the procedure was performed in 148 (83.2%). Among the 30 cases (16.8%) in which the termination of pregnancy did not occur, most were due to noncompliance with the protocol: in 20 cases, the gestational age was incompatible with the date of aggression, the gestational age was > 20 weeks in 6 cases, and in 4 cases the interruption was discontinued at the request of the patient ( Fig. 2 ).

In the history collected by the team, only one patient reported using an effective contraceptive method at the time of violence; in this case, a combined hormonal contraceptive ( Table 1 ).

**Table 1:** Characteristics of women who sought the legal termination of pregnancy service

Characteristics	*n*	%
Age (years old)		
≤14	13	7.3
15 to 19	18	10.1
20 to 29	76	42.7
30 to 39	58	32.6
≥ 40	13	7.3
Skin color		
White	143	80.3
Nonwhite	35	19.7
Scholarity		
Middle school	50	28.1
High school	81	45.5
University education	47	26.4
Occupation		
Employed	113	63.5
Unemployed	20	11.2
Student	45	25.3
Partner		
Married or stable union	16	9.0
No partner	162	91.0
Religion ( *n* = 88)		
Catholic	36	40.9
Spiritism	9	10.2
Evangelicalism	13	14.8
No religion	27	30.7
Other	3	3.4
Primiparous		
Yes	101	56.7
No	77	43.3
Housing		
Florianópolis county	123	69.1
Other cities	55	30.9
Gestational age		
≤12 weeks	146	82.0
12w1d to 16w0d	17	9.6
16w1d to 20w0d	9	5.0
> 20 weeks	6	3.4

Most women had never been pregnant (56.7%), and in 12 cases (6.7%) they were virgins on the occasion of the sexual assault ( Table 2 ).

**Table 2:** Characteristics of the aggression reported by women who sought legal termination of pregnancy ( *n* = 178)

Characteristics	*n*	%
Police report at the time of the assault		
Yes	17	9.6
No	161	90.4
Medical assistance at the time of the assault		
Yes	10	5.4
No	168	94.6
Aggressor		
Unknown	90	50.7
Family	13	7.4
Partner	16	8.8
Ex-partner	25	14.2
Friends	4	2.0
Other acquainted	30	16.9
Number of aggressors		
Unknown	43	24.1
1	134	75.3
> 1	1	0.6
Type of intimidation* (combined or not)		
Physical	105	59.0
Pharmacological	62	34.8
Verbal	28	15.7
Cutting weapon	10	5.6
Firearm	10	5.6
Other	6	3.4
Location of the assault		
Housing	98	55.1
Public street	44	24.7
Party	15	8.4
Unknown	17	9.5
Other	4	2.3

*More than one type of intimidation = 46

The average time between the visit to the hospital and admission for the interruption procedure ranged from the same day of the first visit to up to 28 days, with a median of 3 days and an average of 4.7 days (SD ± 3.2). The total length of hospitalization ranged from 1 to 14 days, with a median of 2 days and a mean of 2.0 days (SD ± 0.7) ( Table 3 ).

**Table 3:** Characteristics of the medical care and procedure for legal termination of pregnancy ( *n* = 178)

Characteristics	*n*	%
Who informed about the right to LTP ( *n* = 54)		
Other health institution	20	37.0
Friend/family	8	14.8
Internet	16	29.6
Police or judiciary authority	4	7.4
Social services	6	11.2
Who referenced the HU-UFSC ( *n* = 98)		
Other health institution	46	47.0
Friend/family	19	19.4
Internet	15	15.3
Police or judiciary authority	10	10.2
Social services	8	8.1
History of attempted unsafe abortion?		
Yes	4	2.2
No	174	97.8
Previously searched another hospital institution?		
Yes	34	19.1
No	144	80.9
Police report at the time of the LTP?		
Yes	24	13.5
No	154	86.5
Outcome		
Pregnancy terminated	148	83.2
Denied	26	14.6
Quitclaim	4	2.2
Method for the LTP		
Misoprostol	4	2.7
Misoprostol + curettage	31	20.9
Misoprostol + MVA	112	75.7
Other	1	0.7
Complications		
Yes (allergy and hemorrhage)	2	1.4
No	146	98.6

Abbreviations: LTP, legal termination of pregnancy; MVA, manual vacuum aspiration.

Table 4 shows the logistic regression analysis between the main characteristics of the cases and the performance or not of the LTP procedure. A gestational age ≤ 12 weeks was the factor associated with a lower chance of the LTP procedure not being performed ( *p* = 0.021). There was an important association between gestational age > 20 weeks and not performing the interruption ( *p* < 0.001). The other study factors were not associated with the performance or not of the LTP.

**Table 4:** Logistic regression analysis between the characteristics of the cases and the performance or not of the procedure among women who sought the service for legal termination of pregnancy due to sexual violence

Characteristics	Performed*	Not performed**	Total	Adjusted OR (95%CI)	*p* -value
	*n* (%)	*n* (%)	*n* (%)		
Age (years old)					
≤ 19	26 (83.9)	5 (16.1)	31 (17.4)	0.97 (0.39–2.72)	0.934
20 to 29	62 (81.6)	14 (18.4)	76 (42.7)	1.28 (0.58–2.91)	0.681
≥ 30	60 (84.5)	11 (15.5)	71 (39.9)	0.88 (0.40–1.97)	0.699
Scholarity					
Middle school	43 (86.0)	7 (14.0)	50 (28.1)	0.81 (0.41–1.89)	0.581
High school	65 (80.2)	16 (19.8)	81 (45.5)	1.51 (0.71–3.55)	0.376
University education	40 (85.1)	7 (14.9)	47 (26.4)	0.87 (0.39–2.35)	0.691
Occupation					
Employed	98 (86.7)	15 (13.3)	113 (63.5)	0.62 (0.31–1.28)	0.133
Unemployed	18 (90.0)	2 (10.0)	20 (11.2)	0.55 (0.29–2.71)	0.451
Student	32 (71.1)	13 (28.9)	45 (25.3)	2.10 (0.81–6.91)	0.068
Gestational age					
≤ 12 weeks	126 (85.1)	20 (66.6)	146 (82.0)	0.41 (0.12–0.88)	0.021
> 12 to ≤ 20 weeks	21 (14.2)	5 (16.7)	26 (14.6)	1.21 (0.42–3.51)	0.771
> 20 weeks	1 (0.7)	5 (16.7)	6 (3.4)	29.93 (3.91–271.5)	<0.001
Skin color, nonwhite				2.01 (0.93–5.89)	0.075
Yes	25 (71.4)	10 (28.6)	35(19.7)		
No	123 (86.0)	20 (14.0)	143 (80.3)		
Married or stable union				0.71 (0.23–3.21)	0.701
Yes	14 (87.5)	2 (12.5)	16 (9.0)		
No	134 (82.7)	28 (17.3)	162 (91.0)		
Reports having a religion ( *n* = 88)				3.41 (0.80–17.21)	0.190
Yes	48 (78.7)	13 (21.3)	61 (69.3)		
No	25 (92.6)	2 (7.4)	27 (30.7)		
Primiparous				0.89 (0.42–1.92)	0.714
Yes	85 (84.2)	16 (15.8)	101 (56.7)		
No	63 (81.8)	14 (18.2)	77 (43.3)		
Metropolitan area				1.60 (0.69–4.21)	0.390
Yes	100 (81.3)	23 (18.7)	123 (69.1)		
No	48 (87.3)	7 (12.7)	55 (30.9)		
Known aggressor				0.91 (0.47–1.99)	0.781
Yes	74 (84.1)	14 (15.9)	88 (49.3)		
No	74 (82.2)	16 (17.8)	90 (50.7)		

Abbreviations: CI, confidence interval; OR, odds ratio.

*Performed: sought the service of legal termination of pregnancy and the procedure was performed

**Not performed: sought the service of legal termination of pregnancy and the procedure was not performed

## Discussion

In the hospital evaluated by the present research, there was a progressive increase in demand for LTP due to sexual violence, notably between 2016 and 2019. This upward trend was also observed in a study performed in the city of Rio de Janeiro, state of Rio de Janeiro, Brazil, during the same period. 19 Data available from the Brazilian Unified Health System Informatics Department (DATASUS) show that, in the period from 2014 to 2019, 7,699 cases of sexual violence were reported in the state of Santa Catarina, with a 70% increase in notifications over these years, which is in line with the growth curve in the present research. 20

The drop in demand for the service in 2020 may be a reflection of the SARS-Cov-2 pandemic, which induced an important change in behavior in the population.

Considering that cases of sexual violence are underreported, a survey conducted in Santa Catarina showed that 6.4% of reported cases of sexual violence resulted in a pregnancy 21 and that there are few services in the state that carry out LTD resulting from sexual violence, 12 the number of cases studied may not match the probable existing demand. The disagreement between these data may be related to the lack of knowledge about women's rights to a LTP.

Many women learn about the right to undergo an LTD and the institutions that offer it after discovering the pregnancy and looking for a health service. 11 In the present study, most women only learned about their rights and about the referral service through an institution, which was contacted after discovering the pregnancy.

The present study showed that most women declared themselves white, without a partner, primiparous, and in the 1 ^st^ trimester of pregnancy, a profile similar to those observed in other studies. 22 23 24 The prevalent age group was 20 to 29 years old, which is in agreement with a study performed in the city of Porto Alegre, state of Rio Grande do Sul, Brazil. 22

Among the cases studied, the highest prevalence was of women who worked and had completed high school. This result was also found in a study in the city of Campinas, state of São Paulo, Brazil. 11 Unlike the present study and the state profile, 21 at the national level, incomplete middle school prevails for victims of sexual violence. 3 4 This difference may be related to the population characteristics of the state of Santa Catarina and to the possibility that women with a higher level of education have more knowledge about their rights.

As for the bond between the victim and the aggressor (50.7% unknown) and the number of aggressors (75.3% with 1), the results obtained corroborate other similar studies. 6 22 23 In 46 cases, > 1 type of intimidation was reported, with a predominance of physical force, in 105 cases (59%). This characteristic corresponds to a national analysis of sexual violence; 4 however, it contrasts with a study performed in Campinas, in which verbal intimidation predominated (35.8%) over the use of physical force (29.1%); however, the present study did not specify the coexistence between types of intimidation. 23

In the present research, in 55.1% of the cases, sexual violence occurred in a residence, which is in line with data on cases of sexual violence in the state of Santa Catarina. 21 At the national level, the prevalence is of sexual violence by an unknown aggressor in public streets. 4 This disagreement may be a consequence of several regional differences in Brazil.

Of the 178 cases analyzed, only 17 (9.6%) filed a police complaint after sexual violence and 10 (5.4%) sought medical care within the first 72 hours. Studies show that in only 10% of rape cases police complaints are filed. 25 This highlights the underreporting and hesitation of women to expose themselves after sexual violence and strengthens the need for an adequate reception in health services by a team capable of listening without judgment.

The report of women who sought other hospitals for LTP before being referred to a reference service (19.1% in the present study) shows the need to expand the services that offer this type of care, both at the state and national level. 26

Of the cases analyzed, 83.2% had termination of pregnancy as an outcome, in agreement with a similar study performed in Porto Alegre, where this number was 90.5%. 22 In a nationwide study of legal abortion, < 50% of women completed the LTP. 26 However, the present study covers services with very different capacities, protocols, and local realities. It also does not clarify the reasons for not performing the procedure.

In Brazilian legislation, there is lack of definition regarding a gestational age limit for LTP resulting from sexual violence. The most recent document that regulates the procedure fails to define a gestational age limit for abortion. 18 Based on Resolution No. 1779/2005 of the Federal Council of Medicine and on norms of the Brazilian Ministry of Health, the HU-UFSC defined as criteria for authorizing the procedure a gestational age < 20 weeks and the compatibility between the date of the occurrence of violence and the gestational age. 8 18

The service analyzed in the present study has a specific multidisciplinary team for LTP, following a well-defined protocol, which ensures performance in accordance with technical standards and reduces the possibility of conscientious objection. This structure is not found in 95% of the services registered to perform the LTP resulting from sexual violence, where care is provided by on-call professionals. 26 Training of the team in welcoming victims is also essential.

The time between looking for the service and hospitalization for the procedure (median of 3 days) and the length of hospitalization (median of 2 days) demonstrate the effectiveness of the service and of the techniques used to terminate the pregnancy.

Regarding the method used for the procedure, WHO recommendation is the association of misoprostol with manual vacuum aspiration (MVA), 27 which was prevalent in the current research with 75.7% of cases. This method also had the highest incidence in a similar study in Porto Alegre/RS with 49.1% of cases. 22 However, MVA is a method of uterine emptying that is not present in part of the services in Brazil that perform LTP. 26

Regarding attempted unsafe abortion, only 2.2% said they had tried it. However, it is noteworthy that this data does not portray the real number of women who tried unsafe abortion, as it only demonstrates those who spontaneously expressed it, as this question was not specifically included in the questionnaires of the service. In Porto Alegre, it was observed that 20.6% of the women stated that they had attempted an unsafe abortion. 22 The practice of unsafe abortion, which is not performed in a medical appropriate environment or by a suitable professional, in Brazil, has an estimated occurrence of > 1 million of these procedures per year. 8 The disagreement between the reported attempts and the actual number can be considered when analyzing that even with the low incidence in studies, postabortion curettage is the third most performed obstetric procedure in public health services in Brazil. 8

There were no maternal deaths in the present study, and in only 2 cases (1.4%) there was a reference to the occurrence of complications, 1 being allergic reaction, and the other, hemorrhaging. These results are compatible with those of a study performed in Campinas, where the rate of complications was 2.3%. 23 Unsafe abortions have a high rate of complications, reaffirming the importance of hospital services that adequately offer the procedure, as well as adequate disclosure of women's rights when faced with a pregnancy with the possibility of provided interruption foreseen by law. 28 29

Gestational age at the time of seeking LTP was the significant factor for not completing the protocol. Pregnancies ≤ 12 weeks were associated with a lower chance of interruption not occurring, whereas cases with a gestational age > 20 weeks were associated with a greater chance of not having interruption. Early search for an LTP service proved to be a protective factor against refusal of the procedure, possibly because the longer the time between the sexual violence and the search for a health service, the greater the probability of an error in the information. Gestational age > 20 weeks places the case outside the protocol of the service and contributes to the denial of the procedure. The reason and the refusal rate for the procedure are not demonstrated and discussed in the available literature.

National policies involving both women victims of sexual violence and LTP resulting from it have evolved and have been regularized in recent decades. However, despite the legislation and technical norms, access to this right still requires certain improvements.

The definition of a protocol and a specialized team for LTP care can offer an effective and resolving service with a minimum risk of complications. The lack of hospitals that offer this service in Brazil and of those that have an adequate staff and protocol is a limitation both for accessing this service and for conducting research on the procedure.

The present research has as limitations its retrospective design and the fact that it is limited to a single institution. Multicenter and prospective studies are needed for a better view of LTP in Brazil.

## Conclusion

The present study has demonstrated the profile of women who seek LTP resulting from sexual violence, which was defined by the regional characteristics of the study. The data demonstrated the effectiveness of the service, with gestational age being the significant factor for resolution.
